# Obstructive sleep apnea in adults

**DOI:** 10.1590/2177-6709.24.3.099-109.sar

**Published:** 2019

**Authors:** Jorge Faber, Carolina Faber, Ana Paula Faber

**Affiliations:** 1 Private practice (Brasília/DF, Brazil).; 2 Universidade de Brasília, Programa de Pós-Graduação em Odontologia (Brasília/DF, Brazil).; 3 Universidade Católica de Brasília, Graduação em Odontologia (Brasília/DF, Brazil).

**Keywords:** Obstructive sleep apnea, Snoring, Orthognathic surgery, Dentistry, Maxillomandibular advancement

## Abstract

**Introduction:**

Obstructive Sleep Apnea and Hypopnea Syndrome (OSAS) is a highly prevalent disease with serious consequences for the patients’ lives. The treatment of the condition is mandatory for the improvement of the quality of life, as well as the life expectancy of the affected individuals. The most frequent treatments provided by dentistry are mandibular advancement devices (MAD) and orthognathic surgery with maxillomandibular advancement (MMA). This is possibly the only treatment option which offers high probability of cure.

**Objective:**

The present article provides a narrative review of OSAS from the perspective of 25 years of OSAS treatment clinical experience.

**Conclusion:**

MADs are a solid treatment option for primary snoring and mild or moderate OSAS. Patients with severe apnea who are non-adherent to CPAP may also be treated with MADs. Maxillomandibular advancement surgery is a safe and very effective treatment option to OSAS.

## RESUMO

**Introdução:**

a Síndrome da Apneia e Hipopneia Obstrutiva do Sono (SAOS) é uma doença muito prevalente e que traz importantes consequências para a vida dos seus portadores. O tratamento da condição é relevante para a melhora do bem-estar geral e da expectativa de vida dos afetados. Os tratamentos odontológicos mais frequentes para a SAOS são os dispositivos de avanço mandibular (DAMs) e a cirurgia ortognática de avanço maxilomandibular (AMM) - essa última, possivelmente, é a única opção de tratamento com alta probabilidade de cura do problema.

**Objetivo:**

o presente artigo faz uma revisão narrativa da SAOS sob a perspectiva de 25 anos de experiência clínica no tratamento da doença.

**Conclusão:**

os DAMs são uma sólida opção de tratamento para o ronco primário e apneias leves ou moderadas. Apneias graves, em pacientes que não se adaptam ou se recusam a usar o CPAP, também podem ser tratadas com os DAMs. A cirurgia ortognática de AMM é uma alternativa segura e muito eficaz de solução da SAOS.

## INTRODUCTION

The Obstructive Sleep Apnea and Hypopnea Syndrome (OSAS) is a disease that brings important negative impacts to people's lives. Its prevalence has increased worldwide^1^ ^,^ ^2^ ^,^ ^3^ as obesity and life expectancy have multiplied. It affects about one in four men and one in ten women.

The diagnosis and treatment of the disease are often neglected, either by the lack of knowledge of dentists and physicians, or by non-adherence of patients to treatment. However, diagnosing and treating is of fundamental importance, and involves multiple specialties cooperating with each other or not. This, to a certain extent, reflects the multifactorial etiology of the disease,^4^ which have anatomical aspects of the airways and jaws,^5^ overweight,^6^ posture during sleep and other factors interacting in the establishment of OSAS.

The treatments can range from weight loss^7^ to maxillomandibular advancement.^8^ The treatment of choice is influenced by the etiology of the problem, but also by personal yearnings - such as the desire or not to change the facial appearance, or the acceptance or not of sleeping with a CPAP mask beside the partner - and socioeconomic characteristics of the patients.

Thus, the aim of this study was to review the literature based on the clinical experience of 25 years of treatment of OSAS in adults. The article mainly focuses on aspects of dental interest.

## BASIC ASPECTS OF SLEEP PHYSIOLOGY

It has long been known that sleep is not a passive phenomenon. While we sleep, the Central Nervous System (CNS) exerts numerous activities, although it maintains a physiological state of loss of vigil consciousness and low responsiveness to external and internal stimuli.

**Figure 1:**
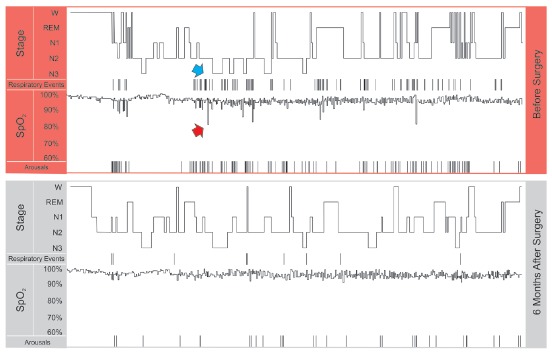
Segments of the polysomnographies of the patient presented in Figure 5, before and after orthognathic surgery. The hypnograms show the patient awake (W), and in the sleep stages of REM, 1 2 and 3. The respiratory events (apneas + hypopneas) are marked by vertical lines, as well as the microarousals (Arousals). Note that prior to surgery there is a certain association between microarousal and respiratory events. Also, blood oxygen saturation (SpO_2_) is associated with respiratory events; the red arrow points to one of the desaturation events that occurred after a respiratory event (blue arrow). The patient had important improvements in number of respiratory events, number of microarousals, blood oxygen saturation, and sleep quality with surgery.

In humans, normal sleep has its own rhythm composed of two distinct states - NREM (non-rapid eye movement) and REM (rapid eye movement) -, during which there is a well ordered and cyclic sequence of wave frequencies that are observed on the electroencephalogram (EEG) during the polysomnographic examination (Fig 1). NREM sleep, also called slow-wave sleep, is divided into three stages that follow each other as sleep consolidates, 1, 2 and 3, the latter being called slow sleep itself, in which there is greater muscle hypotonia. About 90 minutes after the onset of sleep, REM sleep or paradoxical sleep occurs, during which there is presence of dreams, muscular atony and episodes of ocular movements. This NREM + REM sleep composition is called the sleep cycle. In a healthy adult, four or five sleep cycles usually occur during each night, with a higher concentration of stage 3 in the first half of the night and REM in the second.^9^

## OSAS DEFINITION

OSAS is defined by repeated episodes, more than five per hour, of partial or total upper airway obstruction (UAW) during sleep, which lead to airway obstruction (apnea) or reduction (hypopnea) despite maintenance of inspiratory efforts. An apnea event, by definition, must last for at least 10 seconds and is usually associated with hypoxia and sleep fragmentation.^9^ There is no single definition of hypopnea, but it can be defined as a reduction in ventilation of at least 50%, resulting in oxygen desaturation ≥ 4%. Blood oxygen desaturations are a common finding after apnea and hypopnea events (Fig 1).

The Apnea-Hypopnea Index (AHI), which is the mean number of sleep apneas and hypopneas per hour, determines the severity of OSAS. It is considered to be mild between 5 and 14 events, moderate between 15 and 29, and severe when more than 30 episodes occur per hour of sleep.^10^ Other factors such as the oxyhemoglobin desaturation and the percentage of time that desaturation persists throughout sleep also influence the severity of OSAS.

OSAS occurs mainly during REM sleep, in which there is muscle atony, facilitating occlusion of the UAW, and most apnea events culminate with an awakening or microarousal that leads to the return of muscle tonus and cessation of UAW obstruction. As a consequence, it generates sleep fragmentation and superficialization.

The tendency of airway obstruction, or the decrease of its lumen, is often manifested by a noisy vibration of the airway, which is snoring. Most, but not all, patients with OSAS snore. When snoring is an isolated finding, with normal AHI, it can also be termed primary snoring or benign snoring.

## RISK FACTORS

There are many factors associated with the occurrence of OSAS. Anatomical changes that contribute to oropharyngeal space reduction are among the most important of them. Thus, obese individuals with increased neck circumference^11^ and craniofacial alterations - such as increased tongue base, amygdala and uvula^11^- or maxillomandibular deficiencies^12^ are at greater risk for apnea, because there is a reduction in the lumen of the UAW.

Sleeping in the supine position also facilitates the occurrence of apneas due to the posterior repositioning of the tongue by gravitational effect. When alcohol^13^ or other substances are ingested, such as sedatives and myorelaxants, this effect is made even worse by muscle relaxation at both the base of the tongue and the pharyngeal wall.

In addition, smoking is also a risk factor for contributing to UAW dysfunction during sleep, since it tends to promote relaxation of airway muscles, and due to neural reflexes caused by nicotine.^14^ ^,^ ^15^

Women in the menopausal period equate their apnea index with that of men, and it is believed that estrogen and progesterone maintain adequate muscle tone in the premenopausal period.^16^ ^,^ ^17^

## DIAGNOSIS

Snoring is one of the most common predictive signs of OSAS, however, OSAS diagnosis is made by means of polysomnography (Fig 1), a test usually performed in a sleep laboratory. This test occurs at night while the patient sleeps, which allows the monitoring of various physiological and pathological parameters, such as apnea and hypopnea index, oxyhemoglobin saturation, arousals and microarousals, postural changes, distribution of stages of sleep, the electrocardiographic record and the intensity and frequency of snoring.^17^

## CONSEQUENCES

The presence of untreated OSAS is associated with a poorer quality of life and is admittedly an independent risk factor for the development of various clinical diseases and mental disorders.^18^

The cardiovascular and metabolic consequences of OSAS express great concern due to the high degree of lethality before age 65. Systemic arterial hypertension is a very common finding (40% to 60% of cases), and 2/3 of the subjects with acute myocardial infarction had moderate to severe OSA. Severe OSAS increases three to four times the chance of developing cardiac arrhythmias and 3.8 times the chance for stroke. In addition, it also appears to be an important risk factor for increased insulin resistance and diabetes mellitus (types I and II).^10^ ^,^ ^18^

Excessive daytime sleepiness, cognitive impairment, learning deficit, and mental disorders - such as depression and anxiety - may also be associated with OSAS consequences, mainly due to disruption of sleep cycles, which are interrupted at each apnea event. It is estimated that 50% of OSAS patients have depression as a comorbidity.^10^ ^,^ ^18^

The consequences of OSAS impact on not only the individual, but also the society itself; there are indirect consequences of the disease, such as public expenditures, absenteeism, traffic and work accidents, etc.^18^ After adequate OSAS treatment is administered, all these complications have shown great improvement.^10^ ^,^ ^18^

## TREATMENT

The treatment algorithm may involve weight loss, change of posture during sleep, but often therapies of varying degrees of invasiveness are indicated.

## MANDIBULAR ADVANCEMENT DEVICES (MAD)

**Figure 2:**
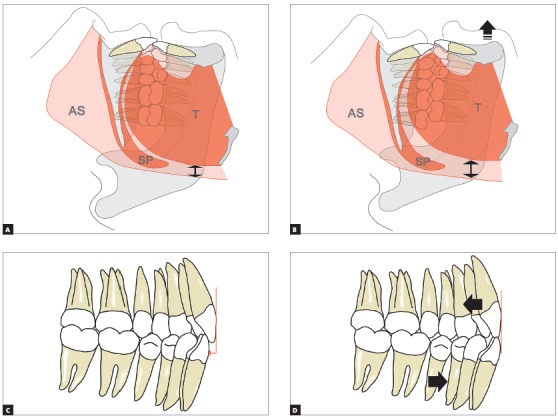
The MAD projects the mandible: A) without projection, B) with projection. Airway space volume (AS) increases (arrows) and/or an improvement in pharyngeal wall tone occurs. This is due to the advancement of the tongue (T) and other para-mandibular soft tissues; even the collapse of the soft palate (SP) undergoes improvement. The overjet (C, in red) decreases over the years (D), by projection of the lower teeth and retraction of the upper ones (arrows).

**Figure 3:**
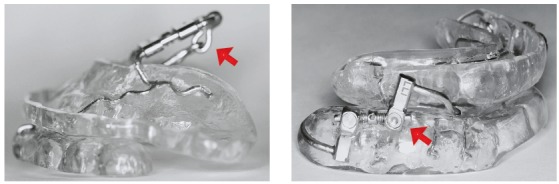
Examples of MADs: different devices can be used to adjust the mandibular position. The arrows point to screws that control the magnitude of the mandibular advancement and allow adjustments.

**Figure 4:**
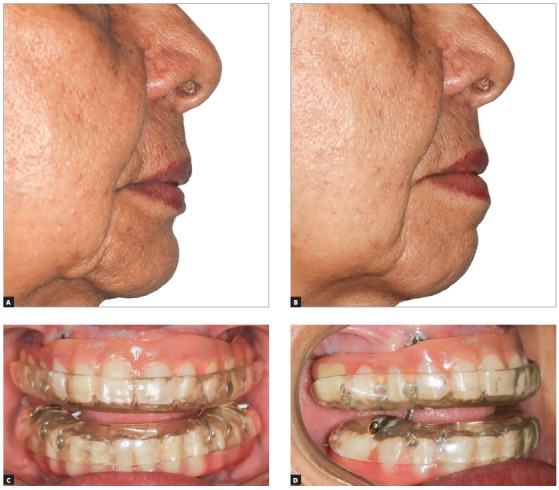
Patient without (A) and with (B) an intraoral device. In this case, the patient has implant-supported dentures. The front (C) and lateral (D) views show the advanced mandible.

The MADs aim to maintain the mandible in an advanced position during sleep^19^ ^,^ ^20^ (Figs 2A, 2B; Fig 3 and Fig 4), promoting a transient increase of the oropharyngeal space during the use of the device and, consequently, reducing obstructions.^21^ They act by pulling the soft tissues anteriorly, especially the genioglossus, genius-hyoids, digastrics and milo-hyoids muscles. The device, however, does not promote a correction of the airways that would result, in the last instance, in cure or permanent improvement - its effect is observed only while the patient is using the device.

Ideally, the patient should be treated by the dentist with individualized and adjustable devices for his/her case. Dental arch impressions or scans are obtained for appliance manufacture. The device should allow a progressive adjustment of the mandibular position. Apparently, less bulky appliances that cover less areas of the mouth are preferred by patients.^22^

### Titration of MAD

When the appliance is installed, it should keep the mandible in a protruded position^20^ ^,^ ^23^ (Fig 2B), previously defined in a bite record. Most people find it difficult to keep their mandible in a very projected position from the first day of treatment because they lack enough joint mobility. Therefore, it is important to start using the appliance gradually, increasing the time of use day by day. It is also important to incrementally adjust the magnitude of mandibular advancement, also called titration.

In the protocol adopted by the authors of the present article, patients are advised to begin using the device gradually. Thus, they receive the recommendation to use the device for about only an hour in the first night, still awake. In the second night, they should increase the use to two hours. In the third, they should start sleeping with the device, but if they wake up in the middle of the night, they should remove it, even if they are feeling very comfortable with the MAD. Thus, they should advance the number of hours of use during sleep until the seventh day, when they should sleep the entire night with the device.

Similarly, the mandible should be advanced gradually by means of adjustments in the MAD. In the protocol described above, the patient initiates the treatment with about 4 to 5 mm of advancement. Thereafter, further advancements of 1 to 3 mm are performed per consultation at 3 to 5-week intervals, until a total mandibular advancement of about 8 to 10 mm is achieved. Some patients tolerate greater advances, others never reach 8 mm.

At the end of the gradual MAD adjustment period, which can take from 3 to 5 months, new polysomnography should be performed to quantify the obtained gains.

### MAD side effects

There are common transitory effects. Most patients report sialorrhea^24^ at the beginning of treatment. Excessive saliva flux usually decreases significantly in just over a month - time necessary for the brain to understand that the object in the mouth is not food and does not need to be digested. However, some patients also report dry mouth.^24^

In addition, patients almost always report some discomfort to their teeth when they remove the device in the morning. This mild pain does not typically impact on chewing or any other function, and tends to disappear still in the morning.

Part of the treated individuals report pain or other symptoms in their TMJs when using the device.^25^ Pain is most often unilateral and may be present from the beginning or develop years after the beginning of the use of the device. In the last scenario, many patients, can establish an association of the pain episode with periods of greater intensity of bruxism, often with an emotional trigger. 

When patients report pain, it is advisable to ask them to stop using the appliance for three to seven days and to prescribe cryotherapy on the affected joint for about ten minutes twice a day. This measure alone significantly controls pain, but the addition of nonsteroidal anti-inflammatory drug may be recommended in the most significant pain conditions. Typically, we prescribe anti-inflammatory medications for up to three days. It is interesting to note that it has been observed that pain is more common in patients who already had pain before starting therapy, whereas joint sounds, clicks, and crepitations may arise with time.^25^

The irreversible adverse effect that occurs in practically every individual who wear a MAD for a prolonged period of time is tooth movement.^26^ ^,^ ^27^ The movements of the teeth tend to decrease the overjet and overbite of the patients (Figs 2C and 2D). This movement may even be positive in cases where the patient has a Class II dental relationship with excessive overbite. However, even when it is negative, for instance when the incisors reach an edge-to-edge relationship or even an anterior crossbite, the MAD treatment can be continued. The risks that OSAS brings out generally outweighs any undesirable side effects in the dentition. However, careful monitoring needs to be done and the option to switch to CPAP or even MMA surgery should always be discussed with the patient.

When the initial negative effects on the dentition start, some patients choose to intercalate the MAD with a CPAP, to mitigate this effect. In the experience of the authors of the present study, it is also common in such cases that patients sleep most nights with a CPAP, but travel or have the first nights with new partners using a MAD.

## MAXILLOMANDIBULAR ADVANCEMENT SURGERY

Surgical modifications of the anatomy of the upper airway have been used as treatment options for patients who do not adhere to CPAP or MAD. There are different surgeries to alter soft tissues directly,^28^ such as laser-assisted uvuloplasty, radiofrequency ablation and others.^29^ However, the most popular soft tissue procedure is uvulopalatopharyngoplasty (UPPP), often nicknamed triple P.

**Figure 5:**
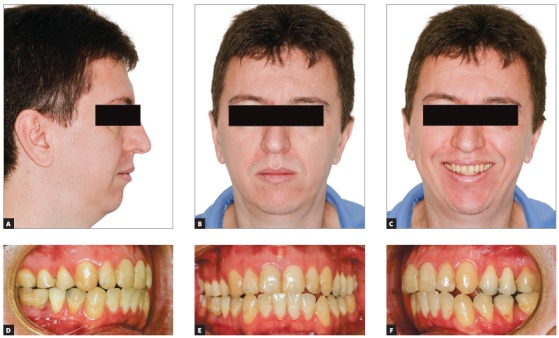
Initial photographs of patients with AHI = 19.7 who underwent surgical orthodontic treatment with the Surgery First protocol. There was a maxillomandibular deficiency (A to C), in addition to a Class II malocclusion (D to F).

**Figure 6:**
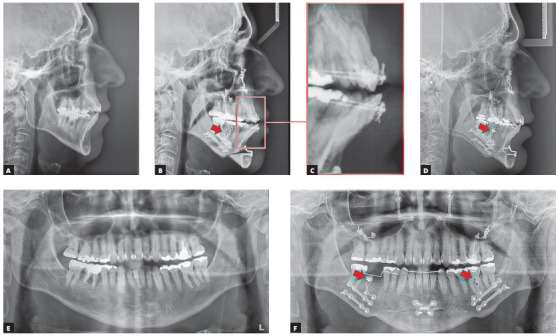
Cephalometric radiographs: initial (A) and immediately after surgery (B). Note that the mandibular advancement was greater than the maxillary advancement, leading to an anterior crossbite (C). This relationship was corrected by means of skeletal anchorage miniplates, which provided the necessary anchorage for lower arch retraction (arrows in B, D and F). The right lower first molar was removed before surgery (E). Panoramic radiographs: initial (E) and immediately after surgery (F).

**Figure 7:**
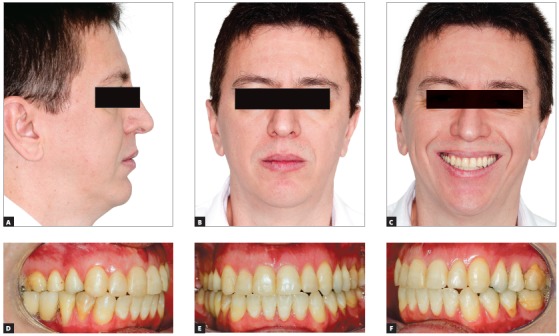
Final photographs of the treatment. A to C show the extraoral views: good aesthetic results were obtained. No counterclockwise rotation of the occlusal plane was performed, to provide good incisor exposure when smiling (C). Good occlusion was achieved at the end of treatment (D to F).

On the other hand, soft tissue modifications can also be obtained by skeletal surgeries, more specifically maxillomandibular orthognathic surgery (Figs 5 to 7).^8^ ^,^ ^12^ ^,^ ^28^ ^,^ ^30^

The case presented in Figure 5 is a male patient, 39 years old, BMI = 26.3, with a major complaint of OSAS. The diagnostic polysomnography revealed that the AHI was 19.7 events/hour. Additionally, he had a facial asymmetry. However, he did not have any aesthetic complaints. The maxillomandibular advancement surgery (Figs 6A and 6B) was performed with a mandibular advancement that exceeded the maxillary advancement. An counterclockwise rotation of the occlusal plane was not performed, in order not to impair the aesthetics of the smile (Fig 7C). The planned outcome of the surgery was obtained and led to an anterior crossbite that was subsequently corrected with the retraction of the lower teeth with miniplates as skeletal anchorage (Figs 6B, 6D and 6F).

The result of the treatment was adequate from both aesthetical (Figs 7A to 7C) and functional perspectives. The treatment led to a good occlusion (Figs 7D to 7F) and, most importantly, a reduction of the AHI to 0.7 events/hour.

The AHI improvement obtained in most cases^28^ with the UPPP has already been shown to be inferior than that achieved with the MMA.^31^ The MMA promotes changes in the airflow dynamics^32^ that benefits patients with significant reductions in AHI.

Patients undergoing MMA surgery are able to perceive the positive change in the airflow immediately after surgery. They often report an easier breathing that they do not remember having experienced before. Typically, improvement in snoring and apnea is noticed on the first night, although significant swelling is already present, both in the airway and externally in the face.

Skeletal surgery must necessarily involve the advancement of the maxilla and the mandible. Adjunctive procedures, such as cervical lipectomy, adenotonsillectomy and others, may also be done during the same surgical procedure.^33^

## DISCUSSION

**Figure 8:**
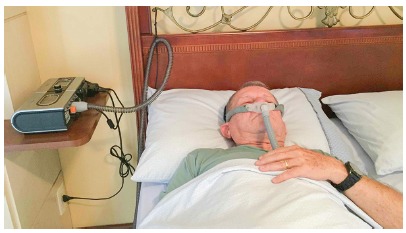
Photograph of a patient with severe OSAS who uses a CPAP with a nasal mask - the CPAP can be seen next to the bed.

One component that restricts OSAS treatment is that treatments in general involve great patient adherence. The use of a MAD or a CPAP every night (Fig 8) requires reasonable discipline: if a 60-year-old English man decides to use CPAP for the rest of his life, which is estimated at 80.2 years,^34^ he will have to sleep with the device for 7,373 nights. Perhaps even more challenging is to lose weight^35^ ^,^ ^36^ and maintain an adequate BMI. Thus, it is not surprising that several patients discontinue treatment over the years, either the CPAP^37^ or MAD^38^ use or the maintenance of adequate BMI. Possibly, at the current stage of development of OSAS treatments, more important than finding the hypothetically optimal therapy for a patient, is to keep the patient in the treatment loop. In other words, when giving up one treatment option, another should be presented so that the patient benefits from being in treatment.

An important restriction to treatment access relies in the communication with patients. The current classification of apnea severity does not convey the actual severity of the disease. From the words used in the stratification of the disease - normal, mild, moderate and severe - two are suboptimal. In English, as in most languages, such as Portuguese or Italian, mild and moderate have an association with unimportant. Many patients with these AHI levels tend to underestimate the problem, and this can be a fatal error. It is for this reason that the authors of the present paper share the opinion that the nomenclature should be changed to normal and grades I, II and III. 

The maxillomandibular advancement (MMA) surgery advantage is that it can be an OSAS cure for many individuals.^8^ This is not excluding the possibility that with advancing age, increased BMI, and changes of other natures, patients cannot have a future worsening of AHI.

MMA beckons as a treatment option that improves the AHI while promoting significant aesthetic and functional breathing gains^32^ as secondary gains. However, it is relatively expensive and comes with the inherent surgical risks. The MMA facial esthetic impact influences the fact that the level of evidence on the association between surgery and AHI reduction is not very high.^39^ In other words, there is a limitation in conducting clinical trials with ideal study designs simply because it is very difficult to randomize individuals to a treatment alternative, such as orthognathic surgery, with relevant impact on patients' self-image.

Patients who are operated on need, in most cases, an associated orthodontic treatment. The preparation for orthognathic surgery in a conventional manner should only be done when a CPAP is used, which in a practical way rarely occurs. There is an urgency to perform the surgery once it is indicated, and unfortunately the conventional preparation for the surgery takes about a year and a half.^40^ Thus, patients who undergo MMA should be treated by means of the Surgery First protocol.^41^ ^,^ ^42^

MMA is often indicated for severe OSAS;^43^ however, patients with mild or moderate AHI can also be successfully treated with this procedure. Obviously, not everyone has an indication for MMA, and even within those who have, only an unknown percentage of patients wishes to undergo such treatment.

If MMA is a great way to treat severe OSAS, MADs are preferentially indicated for mild or moderate OSAS.^23^ Patients with severe OSAS, non-adherent to CPAP and who do not undergo MMA, may also be treated with MADs with relative success. A significant reduction in cardiovascular mortality has been observed when MADs are used in patients with severe OSAS.^44^ However, it is worth noting that this is not the first line of treatment for severe cases, but CPAP or MMA.

MADs are not devoid of limitations. Complaints of sialorrhea, joint and muscle pain, or simply the discomfort of sleeping with the device in the mouth are relatively common. However, the ease of adaptation increases with adequate support from the dentist to encourage the MAD use and to eliminate problems that are within the reach of the professional - for example, smoothing an edge of the device that traumatizes the mucosa.

A very prevalent side effect when using MADs is tooth movement. The muscular traction that provides the desired effects on the pharyngeal soft tissues triggers forces on the dentition. Consequently, the upper teeth tend to suffer a slight retraction, while the lower teeth protrude, resulting in decreased overjet.^26^ These changes may be positive when the patient has a Class II malocclusion; however, they may be undesirable in Class I and III cases. The decision to continue treatment in cases with deleterious effects on the dentition relies on an analysis of each case. A switch to a CPAP or MMA can be considered; however, not following any treatment is not an appropriate option. Thus, if the patient is resistant to adhering to another treatment option, the use of MAD may be continued even if progressive changes in the occlusion still occur.

An important element in the use of MAD is that it is made to be used for an extended period of time, years on end. One should keep this in mind when the device is constructed. Appliances made with vacuum or pressure-formed plates under heat should be avoided. They have low durability and require the making of new appliances constantly.

The prevalence of OSAS is very high. A large portion of the world's population needs treatment and clinicians need to be aware of the existing treatment options. When dentists do not feel able to treat the OSAS patient, they should screen cases and refer them to appropriate treatment.

## CONCLUSION

OSAS is a serious health problem that has important impacts on the quality and life expectancy of affected individuals.

Among the risk factors for the problem are some of direct interest and area of ​​practice of the dentist, such as maxillomandibular deficiencies.

Some risk factors for OSAS are of direct interest and within the scope of dentistry, such as maxillomandibular deficiencies.

MADs are a solid treatment option for primary snoring and mild or moderate OSAS. Patients with severe apnea who are non-adherent to CPAP may also be treated with MADs.

Maxillomandibular advancement surgery is a safe and very effective treatment option to OSAS.
